# Fatigue-free visual perception of high-density super-multiview augmented reality images

**DOI:** 10.1038/s41598-022-06778-4

**Published:** 2022-02-22

**Authors:** Sungjin Lim, Hosung Jeon, Minwoo Jung, Chulwoong Lee, Woonchan Moon, Kwangsoo Kim, Hwi Kim, Joonku Hahn

**Affiliations:** 1 School of Electronic and Electrical Engineering, Kyungpook National University, Daegu, 41566 South Korea; 2 Department of Electronics and Information Engineering, Korea University, Sejong, 30019 South Korea

**Keywords:** Displays, Optoelectronic devices and components

## Abstract

It is well known that wearing virtual reality (VR) and augmented reality (AR) devices for long periods can cause visual fatigue and motion sickness due to vergence-accommodation conflict (VAC). VAC is considered the main obstacle to the development of advanced three-dimensional VR and AR technology. In this paper, we present a novel AR high-density super-multiview (HDSMV) display technique capable of eliminating VAC in wide range. The designed binocular time-sequential AR HDSMV projection, which delivers 11 views to each eye pupil, is experimentally demonstrated, confirming that VAC is eliminated over a wide-range of viewer’s focus distance. It is believed that the proposed time-sequential AR HDSMV method will pave the way for the development of VAC-free AR technology.

## Introduction

Three-dimensional (3D) visual experiences using virtual reality (VR), augmented reality (AR), and mixed reality (MR) display technology have become key components of the fourth industrial revolution. However, visual fatigue and 3D motion sickness have been significant obstacles to the widespread utilization of 3D VR, AR, and MR display technologies^1–5^. The main cause of 3D motion sickness is the binocular disparity of most 3D displays without visual accommodation and results in vergence-accommodation conflict (VAC)^6,7^. As a result, numerous techniques to eliminate VAC based on human visual perception have been studied for 3D displays^8–12^. Despite these efforts, these display technologies are currently unable to eliminate VAC over a wide range of viewer’s focus distance.

Super multiview (SMV) technology is believed to be more practical than other methods for eliminating VAC^13–16^. SMV 3D displays produce VAC-free 3D images by delivering multi-views to each eye of the viewer^17–19^. However, conventional SMV methods have not effectively eliminated VAC over a wide range of viewer’s focus distance due to the low number of views. If the number of views is too small, the SMV 3D contents appear as a set of discretely overlapping images with a lateral shift when the viewer watches the content out-of-focus. The number of views (i.e., the SMV density) in the SMV 3D display is a crucial parameter governing its overall performance and effectiveness in eliminating VAC. We thus recognized the need for an innovative design that could increase the SMV density.

SMV approaches can be classified into spatial-multiplexing and time-multiplexing schemes^20–23^. Takaki presented a 256-view display using multi-projection lenticular displays based on the space-division method, but the SMV density was estimated to be only three views per an eye pupil^24^. Kakeya also demonstrated an SMV display that employed a time-division multiplexing parallax barrier to produce an SMV density of three views^25^. However, to the best of our knowledge, no SMV display has been reported capable of providing more than five views even in the horizontal direction based on an eye pupil diameter of 4 mm^13–30^. To produce VAC-free 3D scenes that are close to the viewer and have a large binocular disparity, a very high SMV density is required.

**Figure 1:**
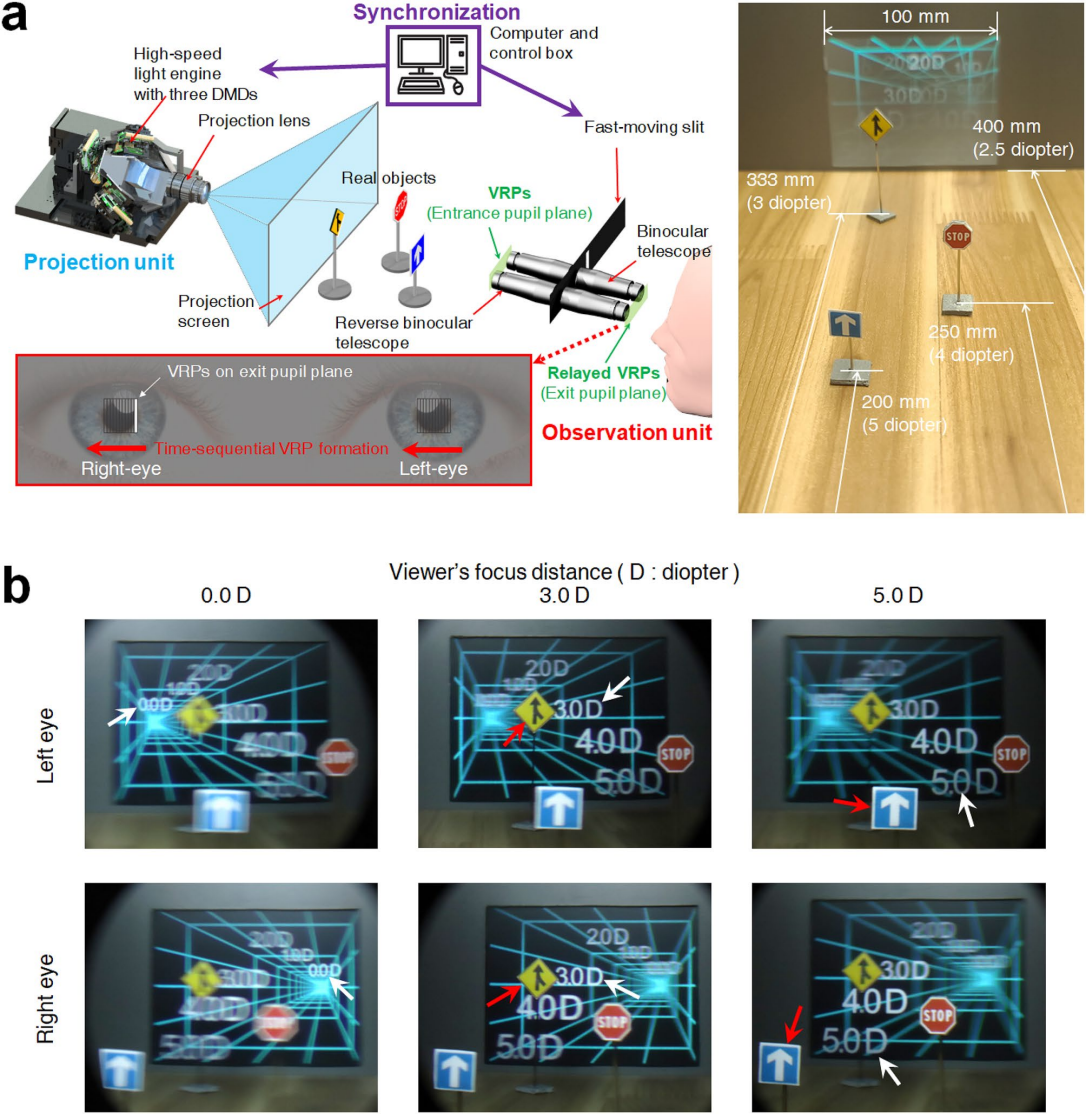
Augmented reality (AR) 3D image free of vergence-accommodation conflict (VAC) over a wide range of viewer’s focus distance. (**a**) Binocular time-sequential high-density super-multiview (BTS HDSMV) projection setup and target object space, and (**b**) binocular SMV-based VAC-free binocular 3D images. The SMV density of eleven views is provided to the viewer’s left and right pupils. VAC is totally eliminated from the 5.0 diopters to 0.0 diopters (see Visualization 1).

In this paper, we investigate a VAC-free AR SMV approach for a wide-range of viewer’s focus distance using a binocular time-sequential high-density super-multiview (BTS HDSMV) projection technique. To experimentally test this BTS HDSMV approach, we devise an AR SMV 3D display system consisting of full-color digital micromirror device (DMD) and the time-sequential shutter. Figure 1a presents the proposed BTS HDSMV projection system and its AR test setup. The projection screen is 2.5 diopters from the viewer, and three traffic signs are placed in front of the projection screen. The merge, stop, and one-way traffic signs are positioned 3.0, 4.0, and 5.0 diopters from the viewer, respectively. In the observation unit of the system, a thin slit moves rapidly in the horizontal direction in front of the viewer’s left and right eyes, allowing them to view a projection image with a real-world scene. The viewpoint provided by the moving slit at a certain instant is referred to as the view reference point (VRP); the synchronized operation of image projection and observation generates multiple VRPs and corresponding multi-view images. At a particular moment, the slit specifies a VRP, while the projection unit of the system displays a corresponding view image on the screen, updating this image as the slit position changes. By controlling the velocity of the slit and the framerate of the synchronized time-sequential projection, the SMV density can be adjusted.

The proposed system configuration produces unprecedented binocular VAC-free 3D images with an SMV density of eleven views. Figure 1b presents an example of the binocular 3D images achieved by the proposed system in the present study. The depth of the real object is indicated by AR images of numbers in a wire grid that indicate the diopter. To show the content behind the screen, we also display objects for a depth of 0.0, 1.0, and 2.0 diopters and the wire-grid object. The 0.0 diopter image represents a focus at infinity, while the 5.0 diopter image has a focus that is 20 cm from the viewer. The in-focus and out-of-focus images are clearly differentiated for the left-eye and right-eye images. The out-of-focus images appear as natural blurred images rather than a discretized overlap of multi-view images. Thus, a natural accommodation effect is obtained for a wide range of viewer’s focus distance. The experimental results show perfect matching of the convergence for the left and right eyes and the corresponding binocular accommodation effect for the entire diopter range.

## Results

### Binocular time-sequential super-multiview projection

**Figure 2:**
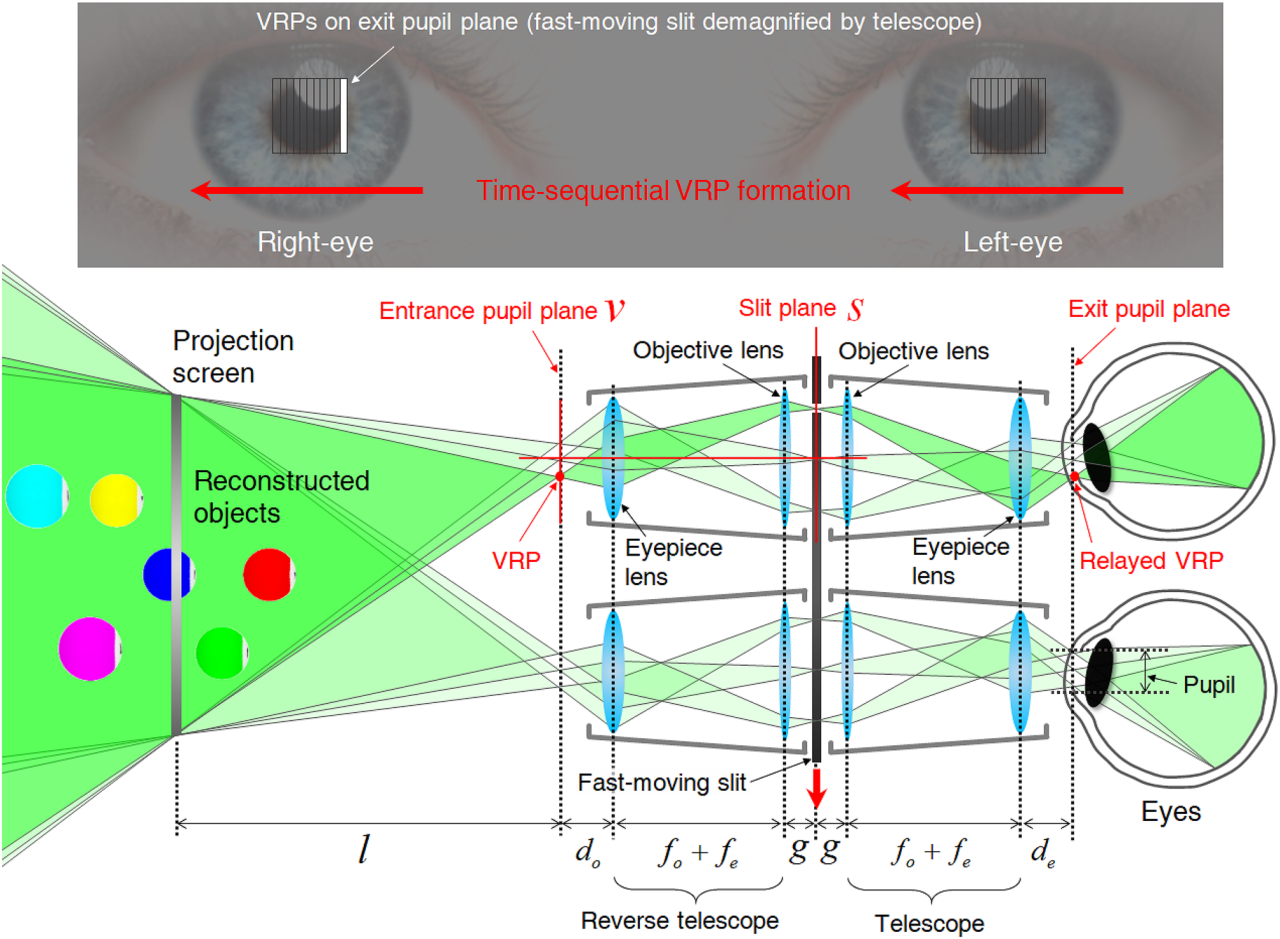
Optical scheme for the proposed BTS HDSMV system. Based on the position of the open slit, VRPs shift and appear at different positions on the exit pupil plane.

In the proposed projection system, a narrow slit with rapid linear movement produces the VRP. Although fast-moving VRP in front of the human eye can be implemented using various methods, we select the simple optical relay design shown in Fig. 2. The observation unit consists of a binocular optical relay with a symmetrical structure consisting of two identical binocular telescopes with a horizontally moving vertical one-dimensional (1D) slit. The purpose of this design is to produce binocular high-density VRP. The slit in the central plane of the symmetric binocular telescope structure creates symmetrical virtual VRPs on the entrance pupil and exit pupil planes. Each VRP is narrow enough and the depth of field is sufficiently long. As the slit moves in a horizontal direction, the viewer’s eye perceives the directional views via the exit pupil plane VRPs. As a result, as viewers vary the focal length of their eyes, it is possible for objects at that distance in SMV content to be in focus. The operation sequence of the proposed BTS HDSMV method is provided in Visualization 2. The focal length of the objective lens $f_{o}$ is much longer than the focal length of the eyepiece lens $f_{e}$. The projection screen is positioned away from the entrance pupil plane by the distance $l$. $d_{o}$ and $d_{e}$ are the distance between the eyepiece lens of the reverse telescope and the entrance pupil plane, and the distance between the eyepiece lens of telescope and the exit pupil plane, respectively. The demagnification of the slit is calculated as

1 $$ v = - s\frac{{f_{e} }}{{f_{o} }}. $$

On the transverse axis, the position of a VRP $v$ is determined by the position of the slit $s$ and the magnification term $- {{f_{e} } \mathord{\left/ {\vphantom {{f_{e} } {f_{o} }}} \right. \kern-\nulldelimiterspace} {f_{o} }}$. The resulting demagnified slit image on the exit pupil plane determine the width of VRP (See Supplementary Information). In addition, while the slit passes through the view synthetic region for each eye, there is no inter-pupillary crosstalk because the left and right-eye are completely segregated from each other by the outer wall of the fast-moving slit.

**Figure 3:**
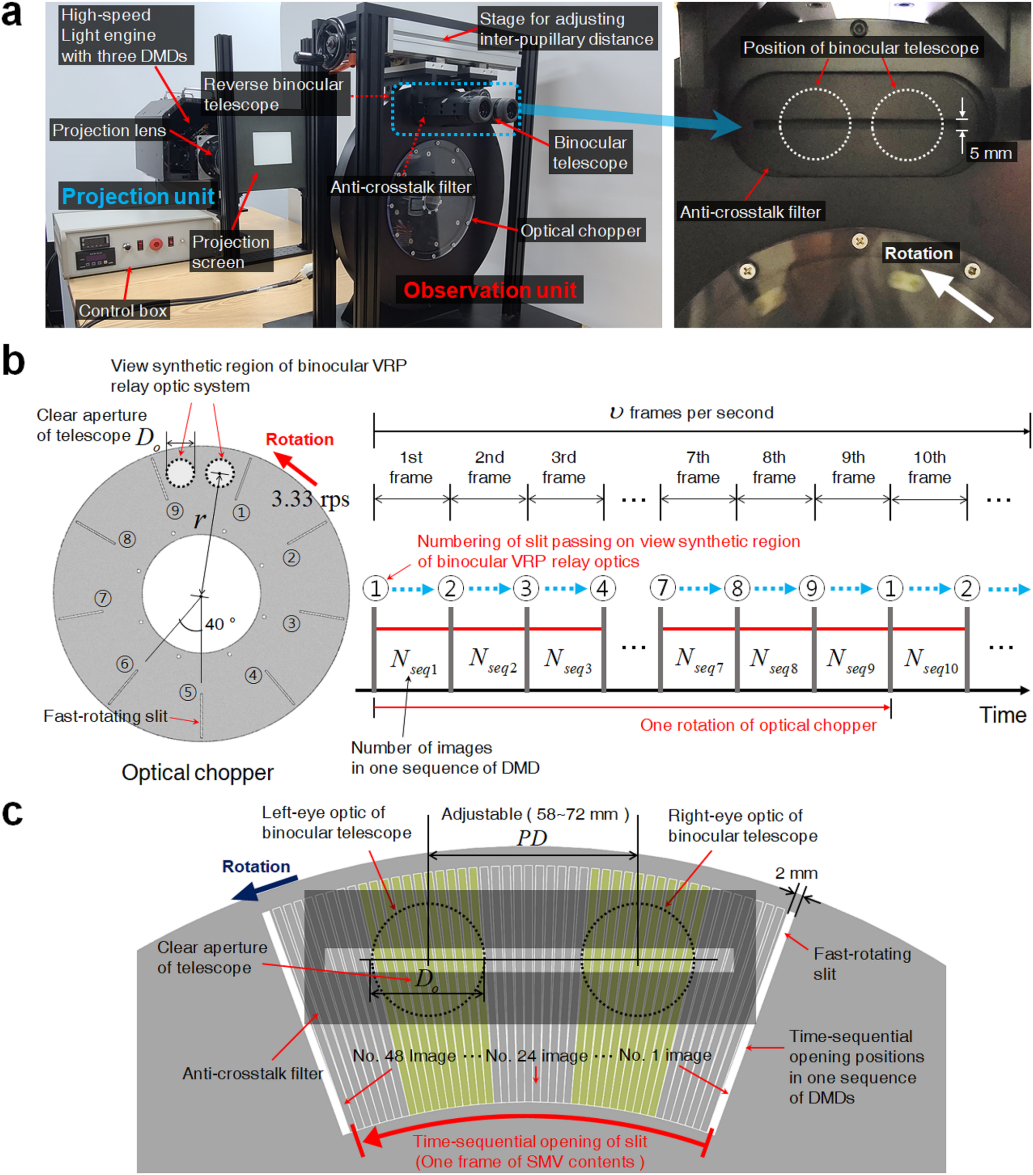
Proposed BTS HDSMV projection system. (**a**) Experimental setup for the projection unit of the devised BTS HDSMV projection. The fast-moving slit in Fig. 2 is implemented using an optical chopper with nine slits. (**b**) Synchronization between the rapidly rotating slit of the optical chopper and the frames of the DMDs. (**c**) Configuration for a single frame of an SMV image.

Figure 3a presents the experimental setup for the proposed BTS HDSMV projection system. The binocular VRP optical relay contains two identical pairs of binocular telescopes (SV32-8, KOWA) with an optical chopper between them. The field of view of the binocular VRP optical relay is about 60 (deg.). The fast-moving slit for the proposed BTS HDSMV system is implemented by rapidly rotating the slit of the optical chopper in the observation unit. The objective focal length $f_{o}$ and the eye-piece focal length $f_{e}$ are 125.6 mm and 15.7 mm, respectively.

The optical chopper has nine slits separated at 40 deg. intervals and the width of these slits is 2 mm. In order to ensure that the VRP motion is linear along the horizontal axis and to prevent inter-view crosstalk caused by the slight inclination of the slit, we place an anti-crosstalk filter on the slit plane. The large diameter rotating optical chopper with the anti-crosstalk filter achieves horizontal linear motion that is similar to the fast-linear moving slit shown in Fig. 2. Additional information about the anti-crosstalk filter is provided in Supplementary Information and Visualization 3. The full-color DMD (V-7001, resolution 1024 × 768 pixels, ViALUX) is operated at a 12-bit color image generation framerate of 1440 Hz, and the optical chopper is synchronized to the DMDs. The distance from the projection screen to the entrance pupil plane $l$ is set to 400 mm. The width and height of the projection screen are 100 mm and 75 mm, respectively. Figure 3b delineates the synchronization between the frames of the DMDs and the slits of the optical chopper. The viewer watches the scene via the very narrow directional ray-bundle arriving through the slits on the chopper. At any instant, only a single slit is placed within the eye-box region including the left and right eyes. If the minimum eye-box length is $D_{o} + PD$, then the interval between the slits on the chopper should be wider than the eye-box length such that

2 $$ D_{o} + PD < \frac{2\pi r}{M}, $$

where $PD$, $D_{o}$, $M$, and $r$ are the inter-pupillary distance, the eye pupil diameter, the number of slits on the optical chopper, and the optical chopper radius, respectively. If Eq. (2) is not satisfied, at least two slits are present in the eye-box region at a given instant, leading to the inter-pupillary crosstalk. In our configuration, the maximum inter-pupillary distance $PD$ is assumed to be 72 mm, and $r$ and $D_{o}$ are set at 170 mm and 32 mm, respectively. According to Eq. (2), we employ nine slits ($M = 9$) in the chopper (Fig. 3b). During a single rotation of the optical chopper, the slit set scans the view synthetic region of the binocular VRP optical relay system nine times. We refer to the time interval between two adjacent slits as one sequence. During a sequence, the DMD projector is programmed to shed $N_{seq}$ view images onto the screen. A single frame of an SMV image is composed of $N_{seq}$ view images. Under these operation condition, the number of views $N$ assigned to an eye pupil area with diameter $D_{o}$, is estimated as follows:

3 $$ N = N_{seq} \frac{{D_{o} }}{2\pi r/M}. $$

The number of view images in one frame $N_{seq}$ is set at 48 (Fig. 3c) so that 48 views do not overlap in the eye-box region. By making the slit narrower, it is possible to increase $N_{seq}$ and vice versa. Among the 48 projected view images in a sequence, only 22 view images in yellow regions (Fig. 3c) are perceivable by two eyes. Therefore, one frame of SMV content has 22 view images and 26 empty images. We set the refresh rate for the SMV content $\upsilon$ to 30 frames per second (fps), which is a level at which human visual perception does not recognize flickering. Because $\upsilon$ is the result of multiplying $M$ by the revolutions per second (rps) of the chopper, the optical chopper is set to have a stable rotation of 3.33 rps. In summary, a chopper with 9 slits rotating at 3.33 rps generates binocular SMV 3D images with a single eye SMV density of 11. The number of views $N$ entering the pupil can be adjusted by changing the number of images in one frame, i.e., $N_{seq}$.

### Binocular HDSMV 3D images

**Figure 4:**
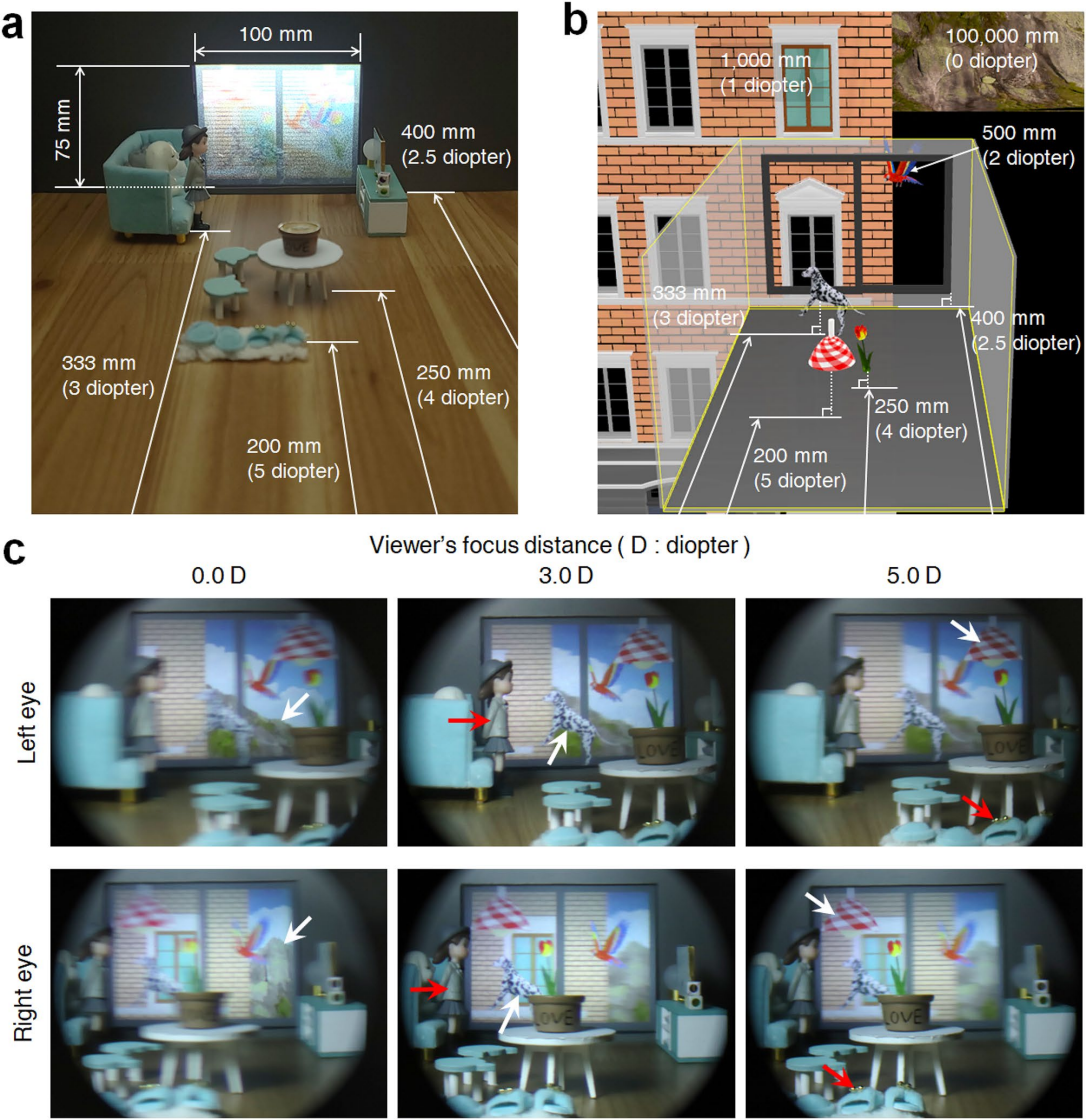
VAC-free AR images. Experimental configuration of (**a**) real objects and (**b**) virtual objects. (**c**) Reconstructed AR images. The AR images are measured by adjusting the focus distance through the binoculars to 0.0, 3.0, and 5.0 diopters.

A gray scale AR image display from 5.0 diopters to 0.0 diopters is presented in Fig. 4.

For 3D content, view images are generated using a 3D modeling tool program, where the virtual camera is set to the VRP. We display virtual objects together with real objects to determine whether the proposed method accurately expresses the depth of the virtual objects. We construct a small room with the screen used as the window (Fig. 4a). The main real objects are slippers, a flowerpot on a table, a doll, and a wall. The distance between these objects and the entrance pupil plane are 5.0, 4.0, 3.0, and 2.5 diopters, respectively. We employ a lamp, a tulip, a dog, a window-frame, a parrot, a building, and a mountain as virtual objects using a 3D contents program as shown in Fig. 4b. The virtual objects are placed at 5.0, 4.0, 3.0, 2.5, 2.0, 1.0, and 0.0 diopters from the entrance pupil plane, respectively. The virtual objects located at 2.5 to 0.0 diopters beyond the window are seen as a part of the landscape outside of the window. The virtual objects at 5.0 to 2.5 diopters match the real objects on the inside of the room.

We determine whether each virtual object is in focus when the corresponding real object at the same depth is in focus by reconstructing the tulip in the flowerpot or reconstructing the dog next to the doll. Figure 4c shows the reconstructed VR images for the viewer’s focus distance of 0.0, 3.0, and 5.0 diopters through the binoculars. When the viewer’s focus distance is 0.0 diopters, the mountain and cloud are in focus and the other objects are blurred. The blurring of these objects depends on their depth. Conversely, if the focus distance is set to 5.0 diopters, the lamp and slippers are in focus and the other objects are blurred. The doll and dog are both in focus when the focus distance is 3.0 diopters. These results illustrate that the proposed method can sufficiently express the depth of objects from 5.0 to 0.0 diopters. A video of the experimental results in Fig. 4c is provided in Visualization 4.

## Discussion

**Figure 5:**
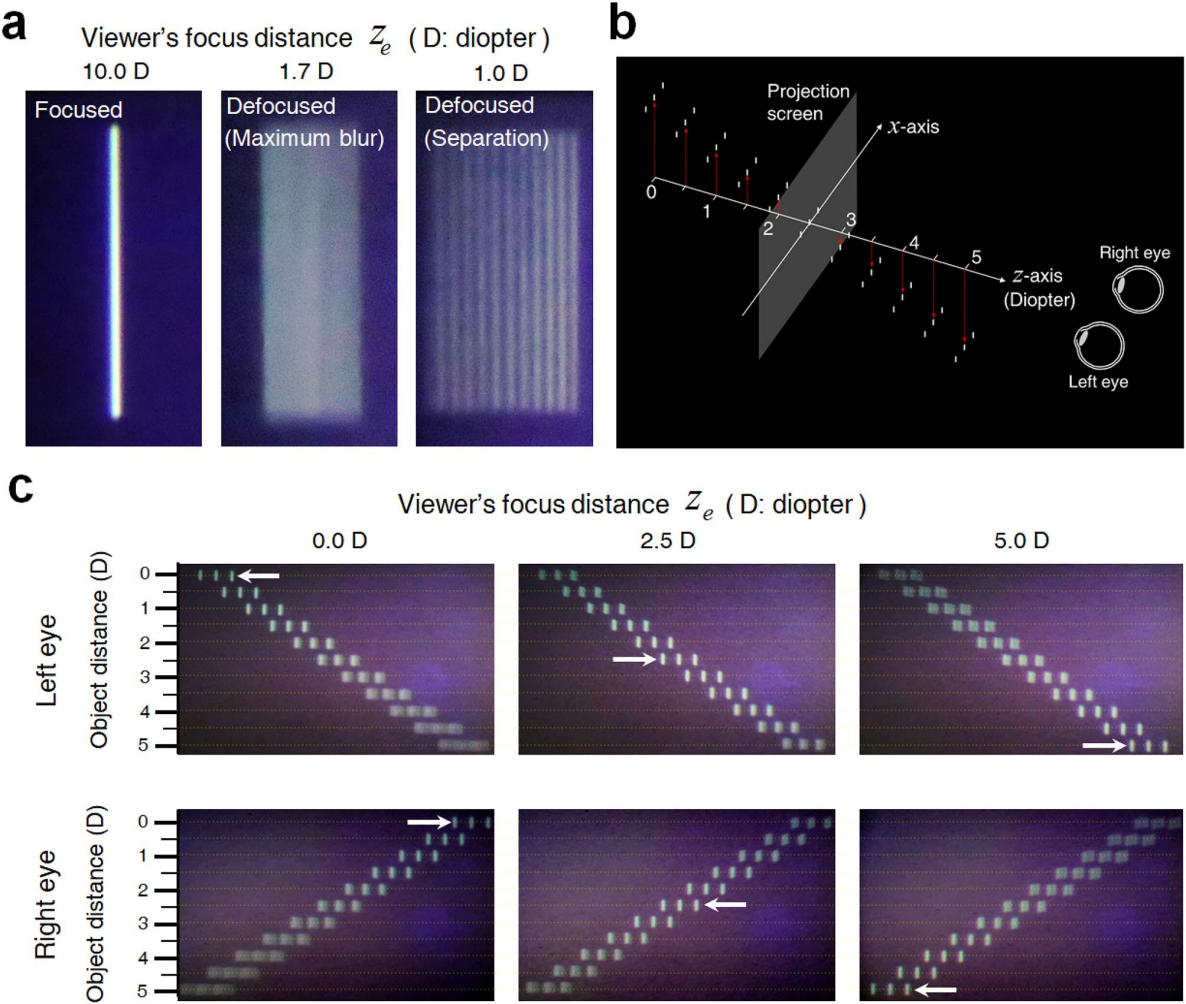
3D images of vertical lines at various depths. (**a**) Monocular 3D images of a vertical line located at $d_{obj}$ = 100 mm depending on the viewer’s focus distance $z_{e}$. (**b**) Experimental configuration of the vertical lines at different depths. Three vertical lines are arranged at object distance $d_{obj}$ from 5.0 to 0.0 diopters, at 0.5-diopter intervals. (**c**) Reconstructed 3D images of the vertical lines at different depths. $z_{e}$ is 0.0, 2.5, and 5.0 diopters.

The accommodation effect for the 3D vertical lines is presented in Fig. 5. Figure 5a presents the right-eye image of a 3D vertical line located 100 mm (10.0 diopters) from the entrance pupil plane. The left panel in Fig. 5a shows the vertical line in focus. The middle panel in Fig. 5a shows the defocused blurred image when the viewer’s focus distance is 1.7 diopters. As the viewer’s focus distance increases, the defocused image separates into 11 vertical lines, as shown in the right panel of Fig. 5a when the view’s focus distance is 1.0 diopter. Each line presents a view image corresponding to 11 VRP positions. The viewer recognizes the dispersed multiple lines instead of a single defocused blurred line. In this case, the viewer may experience the unnatural feeling that the voxel is not blurred but consists of multiple pixels. We distinguish natural-VAC-free and the pseudo-VAC-free zones based on whether the observed 3D image has natural defocus blur or multiple-overlapping blur with the separation of the view images.

To investigate the natural-VAC-free zone for the proposed BTS HDSMV projection system, we performed an experiment to observe vertical lines at several focus distances. As seen in Fig. 5b, three-line objects are arranged from 0.0 diopters to 5.0 diopters with 0.5-diopter intervals. Figure 5c presents the accommodation effect for a single-eye observation with respect to the viewer’s focus distance $z_{e}$. The left, middle, and right panels in Fig. 5c present binocular observation images taken by two cameras with $z_{e}$ of 0.0, 2.5, and 5.0 diopters. When $z_{e}$ is 0.0 diopters, the vertical lines at an object distance of 0.0 diopters are in focus and other vertical lines are blurred. In this case, as expected, the vertical lines located at 5.0 diopters have the largest amount of blur. The natural blurring of the vertical lines is expressed without line separation. When $z_{e}$ is 5.0 diopters, accommodation is perceived at other object distances including 0.0 diopters. The vertical lines at an object distance of 0.0 diopters have the largest blur without the separation and the amount of blur in the vertical line gradually decreases according as the diopter increases. This means that the proposed BTS HDMSV projection system has a potential to display natural 3D objects located from 5.0 to 0.0 diopters. The vertical lines are placed at different depths, but they are all centered horizontally. Therefore, due to binocular disparity, when the viewer watches the vertical lines with either the right or left eye, the vertical line at the point where the vergence does not match appears to shift horizontally in different directions.

Discrimination between the natural-VAC-free zone and the pseudo-VAC-free zone depends on $z_{e}$. The out-of-focus images can be scored according to the amount of defocus blurring. Distinguishing the natural-VAC-free zone from the pseudo-VAC-free zone can be conducted using the two parameters specified in the retina plane: the maximum achievable blur width of one voxel $c_{p}$, which is conceptually defined by the sum of the elemental view image blurring, and the desired natural true blurring of the same voxel, $c_{o}$. $c_{p}$ is a function of $z_{e}$ and the number of VRPs $N$, while $c_{o}$ is the function of $z_{e}$ and the object distance $d_{obj}$. The natural-VAC-free zone is judged by comparing $c_{p} \left( {z_{e} ,N} \right)$ and $c_{o} \left( {d_{obj} ,z_{e} } \right)$ for a particular value of $z_{e}$. When $c_{p} \left( {z_{e} ,N} \right)$ for a given $z_{e}$ is smaller than $c_{o} \left( {d_{obj} ,z_{e} } \right)$ for a voxel at a certain $d_{obj}$, the defocused image of the voxel appears as an overlapped image of separate pieces of elemental view images, as shown in the right panel of Fig. 5a, and the voxel is thus located in the pseudo-VAC-free zone. On the other hand, if $c_{p} \left( {z_{e} ,N} \right)$ is greater than or equal to $c_{o} \left( {d_{obj} ,z_{e} } \right)$, the blurring of the voxel located at $d_{obj}$ can be expressed without separation for a given $z_{e}$. In this case, the voxel is located in the natural-VAC-free zone. Furthermore, the $d_{obj}$ equating to $c_{p} \left( {z_{e} ,N} \right) = c_{o} \left( {d_{obj} ,z_{e} } \right)$ specifies the natural-VAC-free zone in the range of $d_{\min } \left( {z_{e} ,N} \right) \le d_{obj} \le d_{\max } \left( {z_{e} ,N} \right)$. The other area is classified as the pseudo-VAC-free zone. $d_{\max }$ and $d_{\min }$ of the natural-VAC-free zone are a function of $z_{e}$ and $N$.

**Figure 6:**
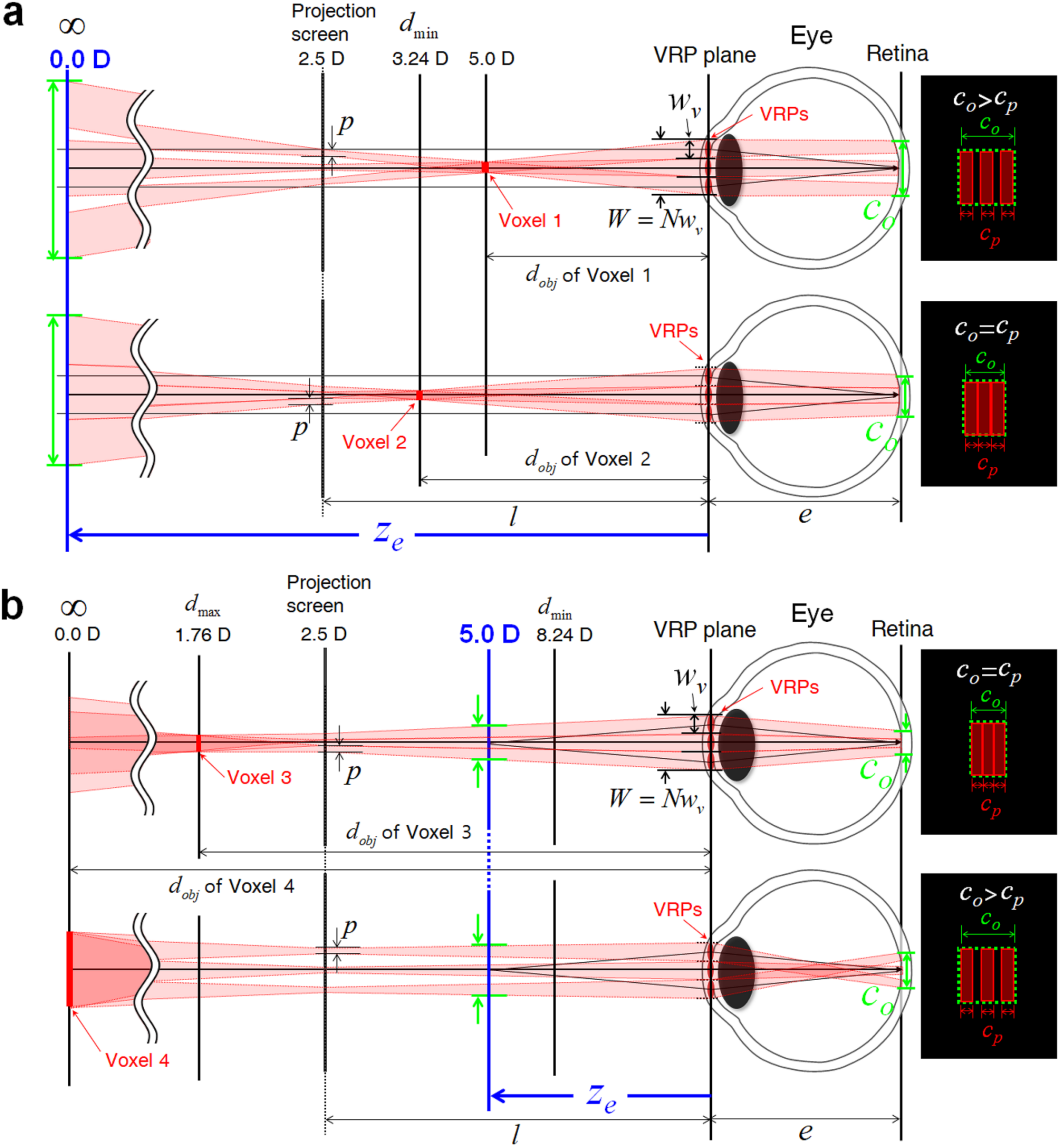
Defocused voxel images of four voxels on the retina plane. Voxels 1, 2, 3 and 4 are positioned at 5.0, 3.24, 1.76 and 0.0 diopters, respectively. (**a**) Images of defocused voxel 1 and voxel 2 at $z_{e}$ = 0.0 diopters. (**b**) Images of defocused voxel 3 and voxel 4 at $z_{e}$ = 5.0 diopters.

Figure 6 presents defocused voxel images of four voxels (voxels 1, 2, 3, and 4) on the retina plane. Without a loss of generality, we assume that the number of monocular VRPs $N$ is 3 in Fig. 6. The comparison of $c_{p} \left( {z_{e} ,N} \right)$ and $c_{o} \left( {d_{obj} ,z_{e} } \right)$ is visualized with $z_{e}$ set to the far-field zone of 0.0 diopters and the near-field zone of 5.0 diopters, respectively, in Fig. 6a and b, in which $c_{o}$ is denoted by a green solid line in the retina image. Each red rectangle on the retina plane is the blurred pixel from the elemental view image and the maximum blur width $c_{p}$ is defined by the sum of the widths of the three rectangles on the retina plane. Voxel 2 is located at $d_{\min }$ with the eye focus at $z_{e}$ = 0.0 diopters, and voxel 3 is located at $d_{\max }$ with an eye focus of $z_{e}$ = 5.0 diopters. Voxels 1 and 4 are outside of the natural-VAC-free zone, while Voxels 2 and 3 are inside this zone. The mathematical representation of $c_{p} \left( {z_{e} ,N} \right)$, $c_{o} \left( {d_{obj} ,z_{e} } \right)$, $d_{\max } \left( {z_{e} } \right)$, and $d_{\min } \left( {z_{e} } \right)$ are derived from the geometric optic analysis of the schematic in Fig. 6. Table 1 summarizes the derived equations. (See Supplementary Information for the full derivation of these equations). The system parameters for the proposed BTS HDSMV system are listed in Table 2, with the numerical values derived from numerical simulations.

**Table 1:** Derived equations for distinguishing the natural-VAC-free zone from the pseudo-VAC-free zone in an SMV display.

Term	Equation	Term definition	Equation number
$c_{p} \left( {z_{e} ,N} \right)$	$c_{p} = N\frac{e}{{z_{e} }}\frac{{w_{v} \left| {l - z_{e} } \right| + pz_{e} }}{l}$	Maximum blur width of a voxel consisting of $N$ views for viewer’s focus distance $z_{e}$	(4)
$c_{o} \left( {d_{obj} ,z_{e} } \right)$	$c_{o} = \frac{e}{{z_{e} }}\frac{{W\left| {d_{obj} - z_{e} } \right|}}{{d_{obj} }}$	Natural true blur width of a voxel with object distance $d_{obj}$ for $z_{e}$	(5)
$d_{\max } \left( {z_{e} ,N} \right)$	$d_{\max } = \frac{{Wlz_{e} }}{{Wl - N\left( {w_{v} \left| {l - z_{e} } \right| + pz_{e} } \right)}}$	Maximum object distance $d_{obj}$ of a voxel consisting of $N$ views in the natural-VAC-free zone for $z_{e}$	(6)
$d_{\min } \left( {z_{e} ,N} \right)$	$d_{\min } = \frac{{Wlz_{e} }}{{Wl + N\left( {w_{v} \left| {l - z_{e} } \right| + pz_{e} } \right)}}$	Minimum object distance $d_{obj}$ of a voxel consisting of $N$ views in the natural-VAC-free zone for $z_{e}$	(7)

**Table 2:** System parameters for the proposed BTS- HDSMV system.

Parameter	Definition	Numerical value
$p$	A pixel width on the projection screen	98 µm
$e$	Distance between the VRP plane and the retina	23.3 mm
$l$	Distance of the projection screen from the VRP plane	400 mm
$N$	Number of monocular VRPs (Number of views entering single eye pupil)	11
$W$	Eye pupil diameter for our modeling	3.7 mm
$w_{v}$	VRP width $w_{v} = {W \mathord{\left/ {\vphantom {W N}} \right. \kern-\nulldelimiterspace} N}$	0.33 mm
$d_{obj}$	Distance of a voxel from the VRP plane	7.0 to 0.0 diopters
$z_{e}$	Viewer’s focus distance	7.0 to 0.0 diopters

**Figure 7:**
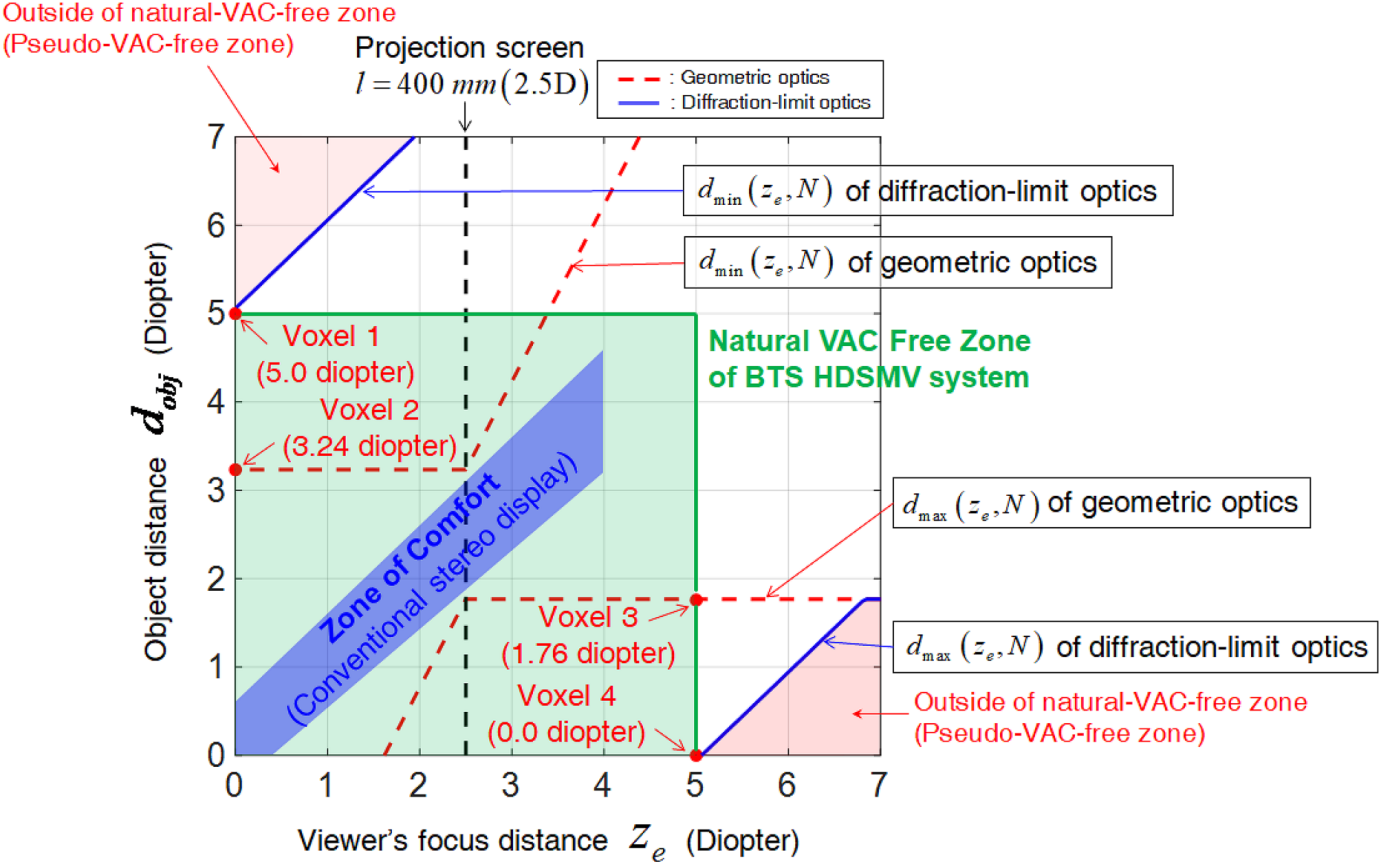
Natural VAC-free zone of the proposed BTS HDSMV system.

Plotting Eqs. (4)-(7) allows the dimensions of the natural-VAC-free zone to be determined (Fig. 7). It is assumed that both eyes can converge on the voxels in all ranges of $d_{obj}$ and $z_{e}$. The red dotted lines represent $d_{\min }$ from Eq. (6) and $d_{\max }$ from Eq. (7), which specify the boundary of the natural-VAC-free zone in Fig. 7.

In practice, we can consider the resolution of one view image on the retina plane to be determined by the VRP width $w_{v}$. The diffraction-limit eye-resolution affects $c_{p}$, and it is possible to expand the depth range of the voxels to the limits specified by the blue lines in Fig. 7 (see Supplementary Information). It is seen that voxels 1 and 4 are not separated because they are located in the natural-VAC-free zone due to the eye-resolution limit. For example, voxels 1 and 4 in Fig. 6 are outside the VAC-free zone and perceived as a separated multi-pixel image according to the geometric natural-VAC-free zone analysis (Eqs. (6) and (7)). However, voxels 1 and 4 are located on the blue lines in Fig. 7, and are thus inside the VAC-free zone. The non-separation of voxels 1 and 4 is verified in the experimental results presented in Fig. 5c. Given that the binocular optical configuration of the BTS HDSMV system has the minimum depth 5.0 diopters in the voxel depth, the finalized VAC-free zone becomes a green rectangular area by in Fig. 6. The green rectangular area is the natural VAC-free zone of the proposed BTS HDSMV system.

The analysis and experimental results indicate that the proposed method widens the natural-VAC-free zone past the limit of previously reported SMV displays by implementing an unprecedentedly high SMV density. The VAC-free zone needs to be compared with the zone of comfort of the conventional stereoscopic 3D displays^8^. This zone is the depth range for 3D objects that supports convergence for both eyes while maintaining viewer comfort. In conventional stereoscopic 3D displays, the depth range of a comfortable 3D image is distributed along the relatively narrow blue zone presented in Fig. 7 because stereoscopic 3D displays provide only binocular disparity. Therefore, in the wide natural VAC-free zone created by the proposed BTS HDSMV method, viewers can see VAC-free binocular 3D images.

## Conclusion

The main purpose of this study is the proof-of-concept validation of the BTS HDSMV projection mechanism. We have verified the principle of the BTS HDSMV method and demonstrated the BTS HDSMV operation with the SMV density of 11 to obtain a natural-VAC-free zone with a wide depth of 0.0 to 5.0 diopters. The numerical and experimental analysis has confirmed the technological feasibility and the potential of BTS HDSMV 3D displays. In the present system, to verify the feasibility, we choose a method of mechanically moving the position of the slit. If the eyepiece is implemented compactly based on electronic fast-moving slit devices such as ferroelectric liquid crystal display, it is expected to make a small form-factor prototype. We plan to research and develop a compact BTS HDSMV near-eye display in the near future.

## Methods

### Hardware development

The BTS HDSMV method requires high-speed display components such as a DMD to allow multiple view images to be rapidly projected onto the screen. The V-7001 DMD with a resolution of 1024 by 768 pixels (ViALUX) was used in the study and it was able to generate a 12-bit color image of 1440 Hz. Three DMDs were used to realize full-color images, with each DMD modulating red, green, or blue (RGB) images. The RGB images were combined using a trichroic prism and projected onto the screen by a projection lens. In the observation unit, SV32-8 telescopes (KOWA) were used. The diameter of the objective lens and the eye-relief were 32 mm and 15.5 mm, respectively. The angular magnification was 8x. We measured the focal length of the telescope lenses; the focal lengths of the objective lens, $f_{o}$ and the eyepiece lens, $f_{e}$ were 125.6 mm and 15.7 mm, respectively. The inter-pupillary distance can be adjusted from a minimum of 58 mm to a maximum of 72 mm. The optical chopper had nine slits and the angle between each slit was 40(deg.). The open width of the slit was 2 mm, and the radius of the optical chopper was 170 mm. The optical chopper was attached to the motor for the movement of the fast-moving slit. We implemented a control box to manipulate the speed of the motor and generated trigger signals to the synchronized projection unit. During one revolution of the motor, the nine slits scanned the view synthetic region of the binoculars. A single frame of SMV content was played back in the projection unit while each slit scanned the view synthetic region once. To reconstruct the SMV content at 30 fps, the motor was driven at 3.33 rps.

## Supplementary Information

Supplementary Information 1.

Supplementary Video 1.

Supplementary Video 2.

Supplementary Video 3.

Supplementary Video 4.

## Data Availability

All the data supporting the findings are available from the corresponding author upon reasonable request.
