# Supplementary material for: Fatigue-free visual perception of high-density super-multiview augmented reality images
Source: Sci Rep. 2022 Feb 22;12:2959. doi: 10.1038/s41598-022-06778-4 (PMC8863894; doi:10.1038/s41598-022-06778-4)
Supplement: Supplementary file 1 — Supplementary Information 1. [file 41598_2022_6778_MOESM1_ESM.docx]

**Fatigue-free visual perception of high-density super-multiview augmented reality images**

**Sungjin Lim^1^, Hosung Jeon^1^, Minwoo Jung^1^, Chulwoong Lee^1^, Woonchan Moon^1^, Kwangsoo Kim^1^, Hwi Kim^2^,** and Joonku Hahn^1^,***

^1^School of Electronic and Electrical Engineering, Kyungpook National University, Daegu, 41566, South Korea

^2^Department of Electronics and Information Engineering, Korea University, Sejong, 30019, South Korea

Corresponding authors: [*jhahn@knu.ac.kr](mailto:*jhahn@knu.ac.kr), **hwikim@korea.ac.kr

# **1. Optical system design**


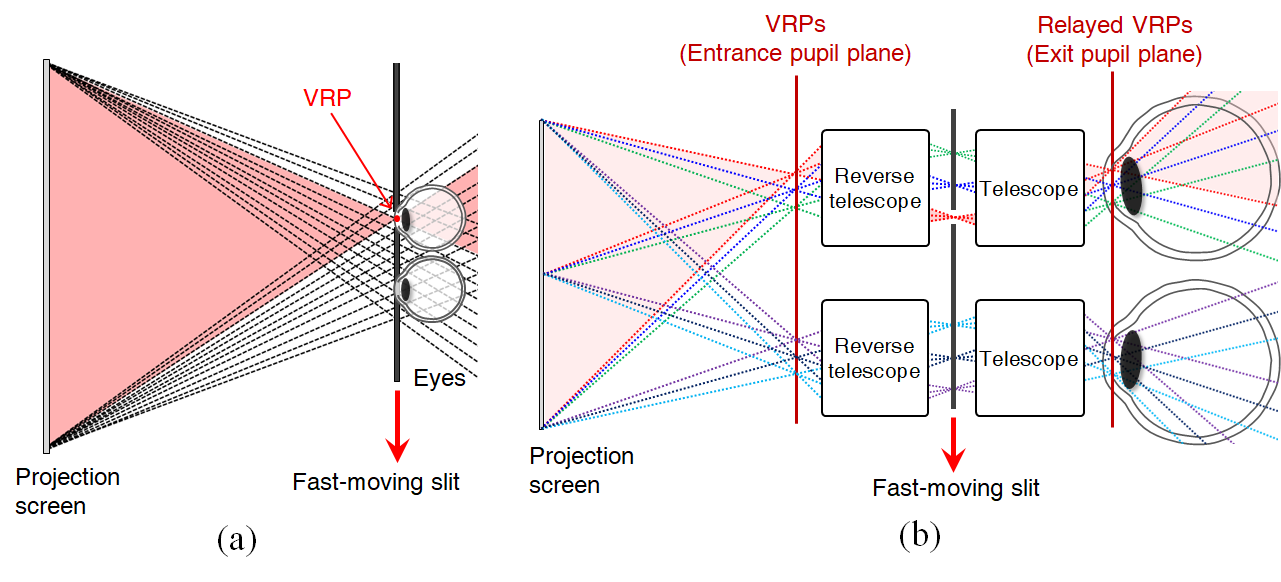


**Figure S1.** BTS HDSMV projection. (a) Ideal condition of BTS HDSMV projection with a fast-moving slit placed on the eye pupil and (b) the implementation of the BTS HDSMV projection with VRP relay optics.

Figure S1a presents an ideal condition of BTS HDSMV projection with a fast-moving slit placed on the eye pupil. The VRPs are determined by the fast-moving slit and the open slit defines the position of the VRP when the slit moves. Assuming that the viewer watches different view images through the fast-moving slit, they can see a 3D image that is free of VAC. The slit needs to rapidly scan the VRP plane and, at a specific VRP, the corresponding view image should be projected onto the VRP synchronously and instantly. The ideal location for a fast-moving slit is in the eye pupil plane. Otherwise, only some information from each view image enters the pupil. Vignetting of some fields of view not only deteriorates image quality, but also reduces accommodation effect. We thus endeavor to design an optical relay for constructing the virtual VRP plane on the eye plane. Figure S1b presents a schematic of the BTS HDSMV projection system. We implement the BTS HDSMV method by configuring the observation unit using a reverse binocular telescope, a binocular telescope, and a fast-moving slit located between the two binocular telescopes. The VRPs that form on the entrance pupil plane are relayed to the exit pupil plane.


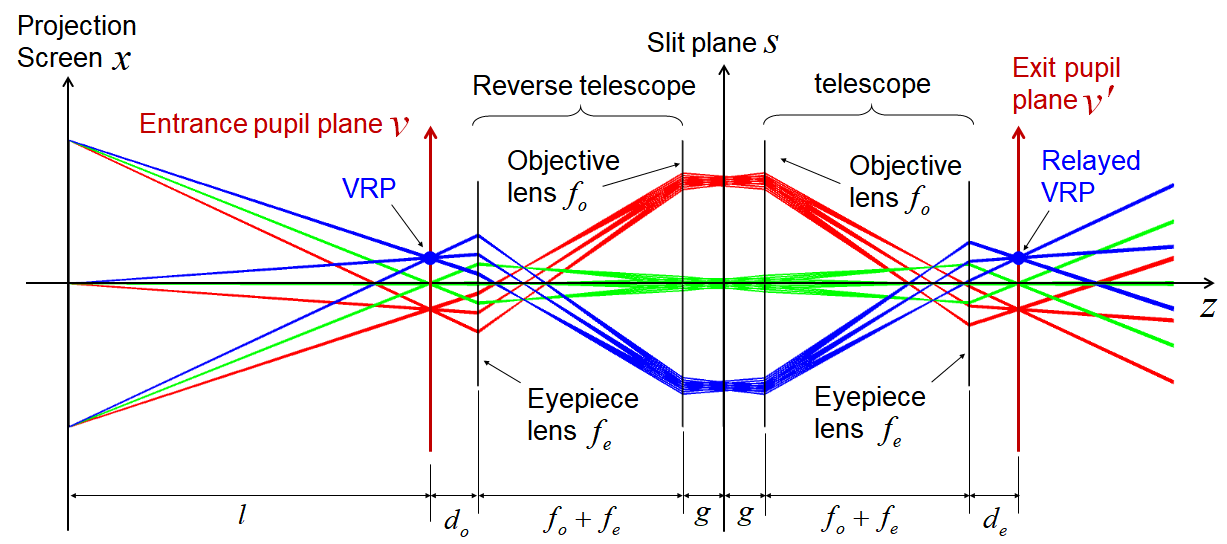


**Figure S2.** Optical relay from the entrance pupil plane to the exit pupil plane in the proposed BTS HDSMV system.

Figure S2 presents the ZEMAX optical layout for a single arm in the proposed binocular relay system. The VRP is placed on the entrance pupil plane, $l$ from the screen. The distance from the entrance pupil plane to the reverse telescope and that from the eyepiece lens of the telescope to the exit pupil plane are $d_{o}$ and $d_{e}$, respectively. $g$ is the gap between the objective lens and the slit plane. The focal length of the objective lens $f_{o}$ is set to be longer than the focal length of the eyepiece lens $f_{e}$. Rays starting from the screen are selected by the slit for a specific VRP, which enter the pupil after passing the correspondent relayed VRP. A ray transfer matrix from the entrance pupil to the exit pupil is obtained by

$\begin{matrix} \left(\begin{matrix} v' \\ k_{v}' \end{matrix}\right)=\left(\begin{matrix} 1 & \lambda d_{e} \\ 0 & 1 \end{matrix}\right)\left(\begin{matrix} -\frac{f_{e}}{f_{o}} & \lambda\left(f_{o}+f_{e}-g\frac{f_{e}}{f_{o}}\right) \\ 0 & -\frac{f_{o}}{f_{e}} \end{matrix}\right)\left(\begin{matrix} -\frac{f_{o}}{f_{e}} & \lambda\left(f_{o}+f_{e}-g\frac{f_{e}}{f_{o}}\right) \\ 0 & -\frac{f_{e}}{f_{o}} \end{matrix}\right)\left(\begin{matrix} 1 & \lambda d_{o} \\ 0 & 1 \end{matrix}\right)\left(\begin{matrix} v \\ k_{v} \end{matrix}\right) \\ =\left(\begin{matrix} 1 & 2\lambda\left(d_{0}-f_{e}-\frac{f_{e}^{2}}{f_{o}}+g\frac{f_{e}^{2}}{f_{o}^{2}}\right) \\ 0 & 1 \end{matrix}\right)\left(\begin{matrix} v \\ k_{v} \end{matrix}\right) \end{matrix}$ (1)

where $k_{v}'$ is the wave number in the transverse direction at position $v'$ on the exit pupil plane. $k_{v}$ is the wave number in the transverse direction at $v$ on the entrance plane, and $\lambda$ is the wavelength. It is assumed that the reverse telescope and the telescope are symmetric such that $d_{o}=d_{e}$. The one-to-one relay condition is given by the (1,2) entry equal to zero;

$d_{o}=f_{e}+\frac{f_{e}^{2}}{f_{o}}-g\frac{f_{e}^{2}}{f_{o}^{2}}.$ (2)

From the (2, 2) entry, the angular magnification is 1. The angle of departure is equal to the angle of incidence. This means that information on the entrance pupil plane are replicated on the exit pupil plane. The relationship between slit plane $s$ and entrance pupil plane $v$ can be explained as follows:

$\left(\begin{matrix} s \\ k_{s} \end{matrix}\right)=\left(\begin{matrix} 1 & \lambda g \\ 0 & 1 \end{matrix}\right)\left(\begin{matrix} -\frac{f_{o}}{f_{e}} & \lambda\left(f_{o}+f_{e}\right) \\ 0 & -\frac{f_{e}}{f_{o}} \end{matrix}\right)\left(\begin{matrix} 1 & \lambda d_{o} \\ 0 & 1 \end{matrix}\right)\left(\begin{matrix} v \\ k_{v} \end{matrix}\right),$ (3)

where $k_{s}$ is the wave number in the transverse direction at position, $s$. From Eqs. (2) and (3), the relationship between $s$ and $v$ is defined as

$s=-\lambda\frac{f_{o}}{f_{e}}\left(d_{o}-f_{e}-\frac{f_{e}^{2}}{f_{o}}+g\frac{f_{e}^{2}}{f_{o}^{2}}\right)k_{v}-v\frac{f_{o}}{f_{e}}=-v\frac{f_{o}}{f_{e}}.$ (4)

Here, $f_{o}/f_{e}$is the magnification factor for $s$. It can be concluded that, under $f_{o}/f_{e}>1$, the interval of the slits in the slit plane is greater than that of the VRPs. The opening width of the slit is demagnified by the reverse telescope and imaged onto the entrance pupil plane, meaning that the demagnified slit determines the width of the VRP.

# **2. Anti-crosstalk filter**

In the experimental setup, we devise a simple anti-crosstalk filter on the exit pupil plane (Fig. S4). Crosstalk, which is used as a figure of merit (FOM) for SMV 3D image quality, is a measure of the indistinguishability of views within the pupil. In the SMV system, the crosstalk deteriorates the 3D image resolution and 3D depth cues. The proposed BTS HDSMV projection system has no inter-pupillary crosstalk. However, in the practical implementation of our prototype, the moving slit is set up on a rotating optical chopper, and the circular path of the slit causes inclined VRPs leading to some inter-view crosstalk. On the exit pupil plane of the right eye taken using long exposure mode of the camera, showing that Fig. S3a presents an image of the VRPs. The VRP patterns are slightly inclined towards the right. The width of the exit pupil plane is 4 mm, and the width of each bright line is about 0.27 mm. There are 11 bright VRP bright lines in the pupil. In a similar manner, the VRPs are slightly inclined towards the left for the left eye.


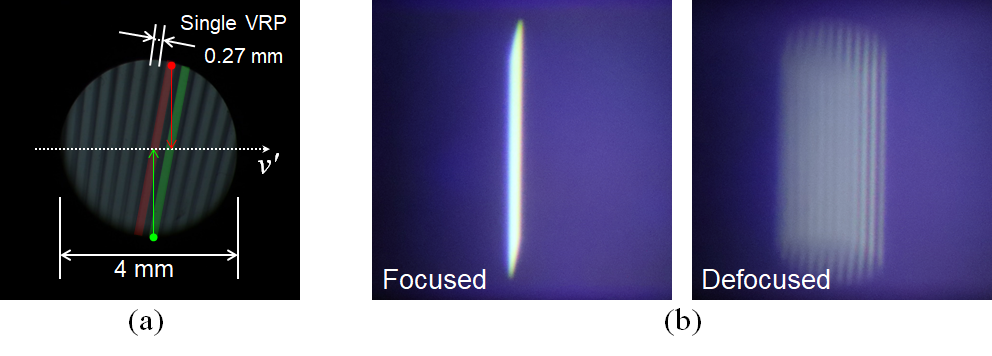


**Figure S3.** Generation of inclined VRPs. (a) Captured image of relayed VRPs in the exit pupil plane and (b) 3D images of the vertical line located at 100 mm.

Although the ideal BTS HDSMV projection system is configured to provide the horizontal parallax so that the adjacent VRPs do not invade each other’s area in the horizontal direction, the adjacent inclined VRPs invade the area of the adjacent VRP in the horizontal direction causing inter-view crosstalk (Fig. S3a). Figure S3b presents 3D images


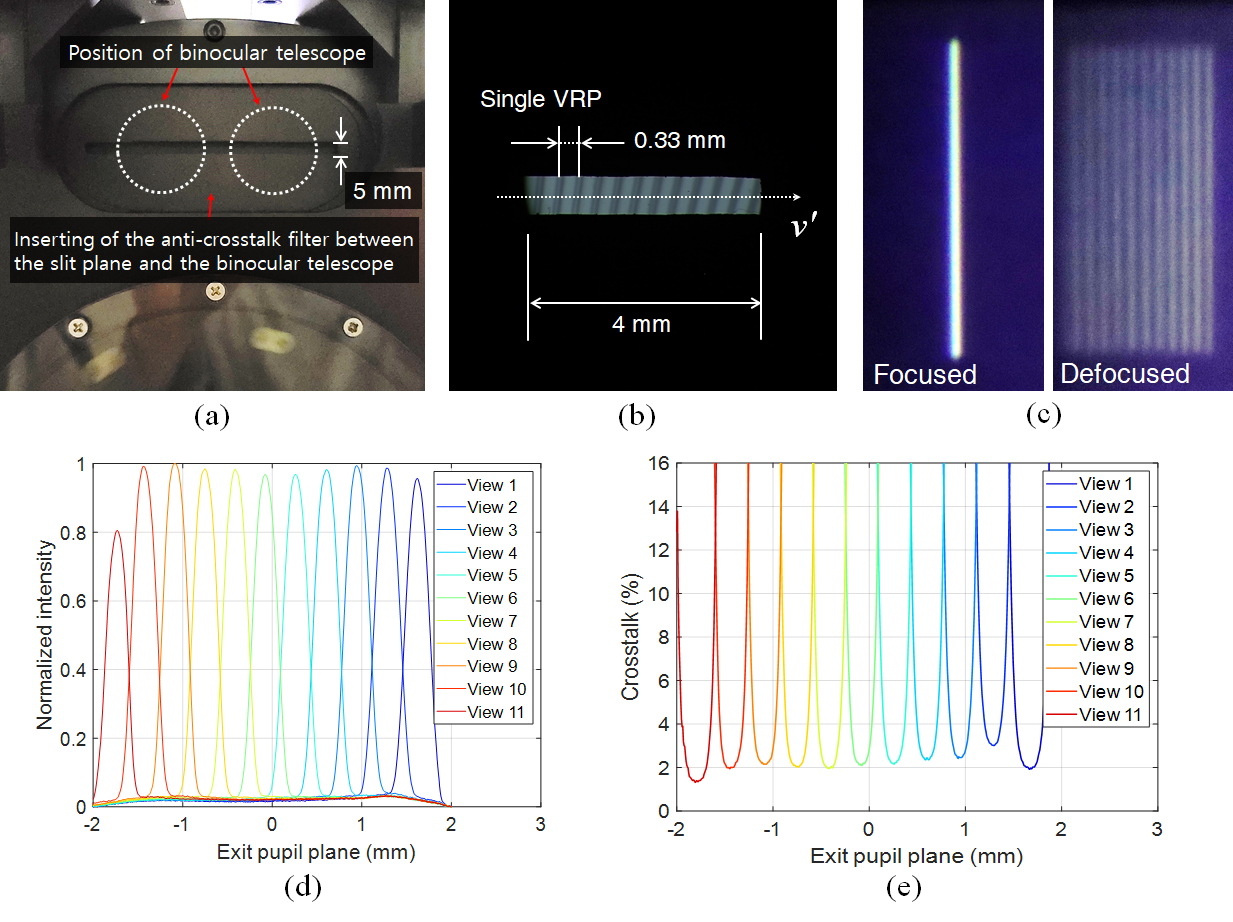


**Figure S4.** Inserting an anti-crosstalk filter between the optical chopper and the binocular telescope. (a) Experimental setup for the anti-crosstalk filter, (b) captured image of the relayed VRPs on the exit pupil plane with the anti-crosstalk filter, and (c) 3D images of the vertical line at 100 mm. (d) Intensity distribution and (e) crosstalk evaluation of VRPs on the exit pupil plane.


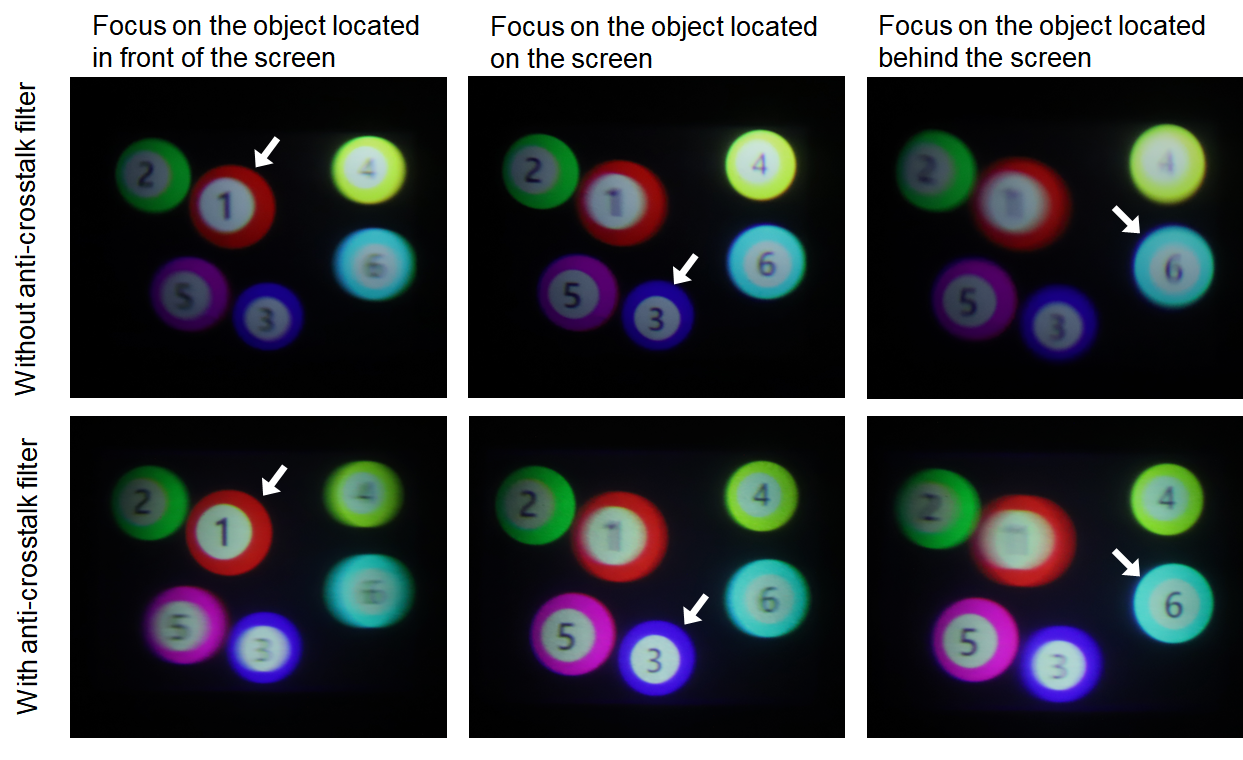


**Figure S5.** Comparison of 3D contents in the presence and absence of the anti-crosstalk filter (see Visualization 3).

of a vertical line when the line is in focus or out-of-focus. The vertical line is positioned 100 mm from the entrance pupil plane. We investigate whether the leaning VRPs affect the blur direction of the defocused and in-focus line image (Fig.3b). It is found that the exact depth of the 3D object is not able to be expressed due to the inter-view crosstalk caused by the inclined VRPs.

In order to reduce the inclination of the VRPs, we insert an anti-crosstalk filter between the fast-rotating slit plane and the binocular telescope (Fig. S4a), which reduces the vertical width of the VRPs and prevents the crosstalk. In the experimental setup, the optimal opening height of the anti-crosstalk filter is set at 5 mm by comparing the averages of the crosstalk. The exit pupil plane with the anti-crosstalk filter is captured in Fig. S4b; the width of a single VRP is about 0.33 mm in the horizontal direction. Figure S4c shows focused and defocused images of the same vertical line shown in Fig. S3b. The 3D images of the vertical line are clearly improved with the addition of the anti-crosstalk filter (Fig. S4c). The focused vertical line is rectangular in shape and in clear focus. In contrast, the defocused vertical line is blurred in the horizontal direction and 11 lines are clearly observed. In addition, the intensity distribution of the VRPs on the exit pupil plane with the use of the anti-crosstalk filter is presented in Fig. S4d and the resulting crosstalk is presented in Fig. S4e. The minimum crosstalk of the 11 VRPs are 1.31%, 1.94%, 2.15%, 2.02%, 1.97 %, 2.09 %, 2.15 %, 2.35 %, 2.41 %, 3.00 %, and 1.92 %. The average of the minimum crosstalk is 2.12%, verifying that the proposed BTS HDSMV projection system produces SMV images with a high VRP density and low inter-view crosstalk. We also reconstruct 3D objects with and without the anti-crosstalk filter (Fig. S5), and the results are measured using the right eye relay. Six billiard balls are numbered from 1 to 6 in order of the distance from the entrance pupil (see Fig. 2 in the main manuscript). The No. 3 billiard ball is located at the same position as the screen. The No. 1 and 2 billiard balls are positioned in front of the screen and the No. 4, 5, and 6 billiard balls are behind the screen. The No. 1, No. 3, and No. 6 billiard balls are put in focus sequentially. Overall, the 3D images are improved with the use of the anti-crosstalk filter, as illustrated by a comparison of the No. 1 and No. 6. Billiard balls. In particular, with the anti-crosstalk filter, the No. 6 billiard ball exhibits better focus and the No. 1 billiard ball has better horizontal blur.

# **3. Conditions for eliminating vergence-accommodation-conflict**

The overall purpose of the SMV display is to reduce VAC via the viewer’s focus distance, thus it is important to evaluate the depth of 3D content that the SMV display is capable of presenting without VAC. The zone of comfort is the region where the viewer can watch 3D images without discomfort on a conventional stereoscopic display (Fig. S6a) [1]. In conventional stereo displays, the depth range for 3D objects required to produce comfortable 3D images is narrow because only binocular disparity is utilized (Fig. S6b). In contrast, with an SMV display, both eyes of the viewer observe a multi-view image, and the comfortable depth zone is broadened as shown in Fig. S6a.

We distinguish the depth zones into the natural-VAC-free zone and the pseudo-VAC-free zone according to whether the observed 3D image possesses the natural defocus blur or the multiple-overlap blur. The natural and pseudo-VAC-free zones are expressed by two parameters from the retina plane: the maximum achievable blur width $c_{p}$ of one voxel, which is defined by the sum of the elementary image blur, and the desired natural blur $c_{o}$ of the same voxel. The width of single VRP $w_{v}$ is the width of the imaged slit on the pupil. Afs shown in Fig. S6a, when the eye stares at a position in front of the screen $\left(z_{e}\leq l\right)$, the width of the blurred pixel $\chi_{near}$ at $z_{e}$ is

$\chi_{near}=\frac{w_{v}\left(l-z_{e}\right)+pz_{e}}{l}.$ (5)

Similarly, when the eye stares behind the screen $\left(z_{e}\geq l\right)$(Fig. 7b), the width of blurred pixel $\chi_{far}$ is defined as

$\chi_{far}=\frac{w_{v}\left(z_{e}-l\right)+pz_{e}}{l}.$ (6)

The maximum achievable blur width $c_{p}\left(z_{e},N\right)$ of one voxel on the retina is obtained from Eqs. (5) and (6) with the consideration of the magnification by the eye:


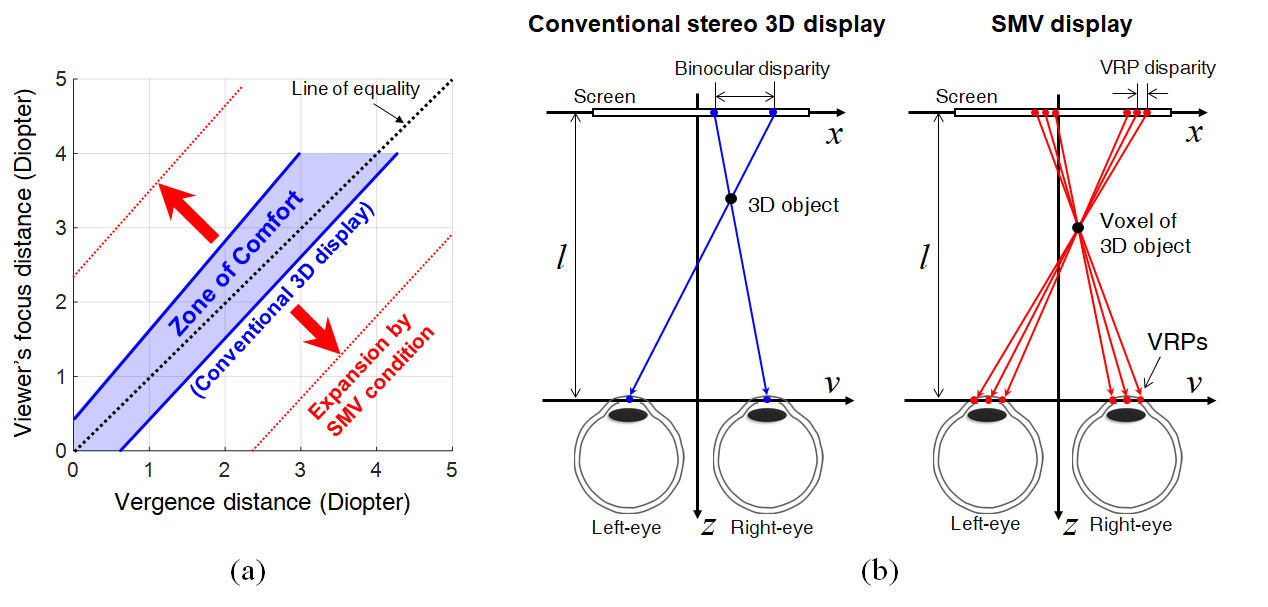


**Figure S6.** Comparison of an SMV display with a conventional stereo 3D display. (a) Relationship between the vergence distance and the viewer’s focus distance, depicting the zone of comfort. (b) Optical schemes for a conventional stereoscopic 3D display and an SMV display.


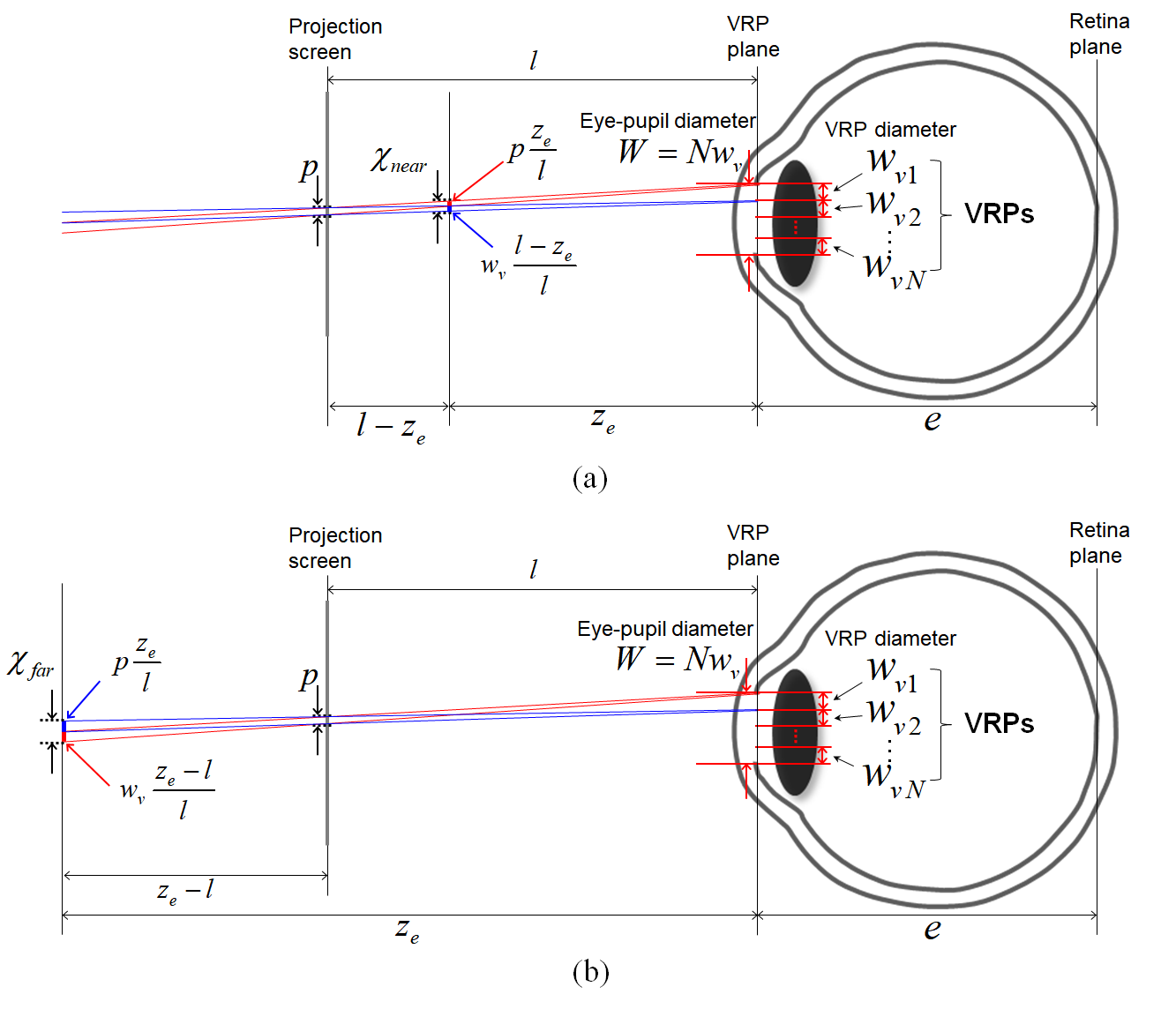


**Figure S7.** Geometry employed to obtain the maximum achievable blur width $c_{p}$ of one voxel. (a) Blur width when the eye stares at the front of the screen $\left(z_{e}\leq l\right)$ and (b) the blur width when the eye stares behind the screen $\left(z_{e}\geq l\right)$.


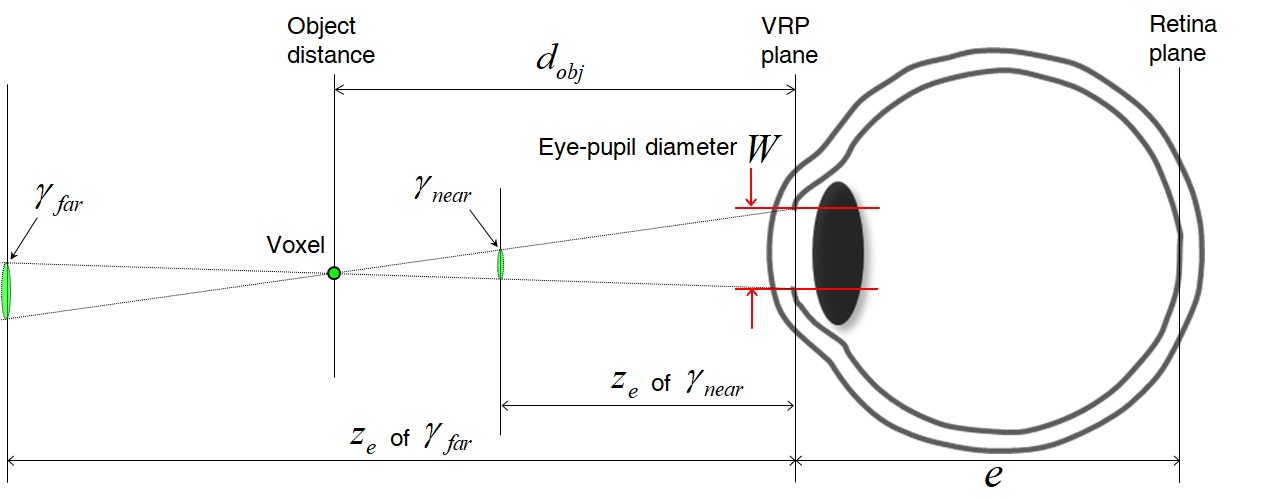


**Figure S8**. Geometry employed to calculate the desired natural true blur width of one voxel $c_{o}$.

$c_{p}\left(z_{e},N\right)=N\frac{e}{z_{e}}\frac{w_{v}\left|l-z_{e}\right|+pz_{e}}{l}.$ (7)

Equation (7) indicates the maximum achievable blur width of one voxel with *N* views at a given $z_{e}$. The pupil diameter $W$in the SMV system is assumed to the product of the VRP width $w_{v}$ and $N$, i.e., $W=Nw_{v}$.

Figure S8 depicts the geometry used to calculate the desired natural true blur width of one voxel $c_{o}$. The voxel is positioned at object distance $d_{obj}$ from the VRP plane. When the viewer focuses at $z_{e}$, the sizes of the natural blurred voxel in the front and behind $d_{obj}$ are denoted as $\gamma_{near}$ and $\gamma_{far}$, respectively,

$\gamma_{near}=\frac{W\left(d_{obj}-z_{e}\right)}{d_{obj}},$ (8a)

$\gamma_{far}=\frac{W\left(z_{e}-d_{obj}\right)}{d_{obj}}.$ (8b)

Thus, $c_{o}$ is a function of $d_{obj}$ and $z_{e}$ given by

$c_{o}\left(d_{obj},z_{e}\right)=\frac{e}{z_{e}}\frac{W\left|d_{obj}-z_{e}\right|}{d_{obj}}.$ (9)

Equation (9) specifies the natural blur width of a voxel (Fig. S8). $c_{p}$is a function of $z_{e}$ and the number of VRPs $N$ and is independent of $d_{obj}$, while $c_{o}$ is the function of $z_{e}$ and $d_{obj}$ . The natural-VAC-free zone is identified by comparing $c_{p}\left(z_{e},N\right)$ and $c_{o}\left(d_{obj},z_{e}\right)$ for a given $z_{e}$. When $c_{p}\left(z_{e},N\right)$ is smaller than $c_{o}\left(d_{obj},z_{e}\right)$ $\left(c_{p}<c_{o}\right)$ for a voxel at a certain depth $d_{obj}$ , the defocused image of the voxel appears as an overlapping image of separate elemental view images (Fig. S5c), and the voxel is classified as being in the pseudo-VAC-free zone. On the other hand, if $c_{p}\left(z_{e},N\right)$ is greater than or equal to $c_{o}\left(d_{obj},z_{e}\right)$ $\left(c_{p}\geq c_{o}\right)$, the blur of the voxel located at $d_{obj}$ can be expressed without separation. In this case, the voxel is located in the natural-VAC-free zone. Furthermore, the borderline of the natural-VAC-free zone is specified at $c_{p}\left(z_{e},N\right)=c_{o}\left(d_{obj},z_{e}\right)$, representing the range of $d_{obj}$ in $d_{\min}\left(z_{e},N\right)\leq d_{obj}\leq d_{\max}\left(z_{e},N\right)$. The other area is referred to as the pseudo-VAC-free zone. The maximum $d_{\max}$ and the minimum $d_{\min}$ of the natural-VAC-free zone are a function of $z_{e}$ and $N$;

$d_{\max}\left(z_{e},N\right)=\frac{Wlz_{e}}{Wl-N\left(w_{v}\left|l-z_{e}\right|+pz_{e}\right)},$ (10a)

$d_{\min}\left(z_{e},N\right)=\frac{Wlz_{e}}{Wl+N\left(w_{v}\left|l-z_{e}\right|+pz_{e}\right)}.$ (10b)

If the VRP width $w_{v}$ is maintained, an increase in $N$ expands the natural-VAC-free zone.

Figure S9 presents the estimation result for the natural-VAC-free zone in the BTS HDSMV projection system by using Eqs. (10a) and (10b). The blue area represents the natural-VAC-free zone without separation depending on $z_{e}$. Outside of the blue area is the pseudo-VAC-free zone with separation. For example, voxel 1 in Fig. S9 is located 5.0 diopters from the VRP plane and $z_{e}$ is 0.0 diopter. In this case, $c_{o}\left(d_{obj}=0.0\text{D, }z_{e}=5.0\text{D}\right)$ is larger than $c_{p}\left(z_{e}=0.0\text{D,} N=11\right)$, and voxel 1 is recognized as separate elemental view images when the eye stares at a distance of 0.0 diopters. Similarly, voxel 4 located at 0.0 diopters is also recognized as separate elemental view images when the eye gazes at 5.0 diopters because $c_{o}\left(d_{obj}=5.0\text{D, }z_{e}=0.0\text{D}\right)$ is larger than $c_{p}\left(z_{e}=5.0\text{D,} N=11\right)$.


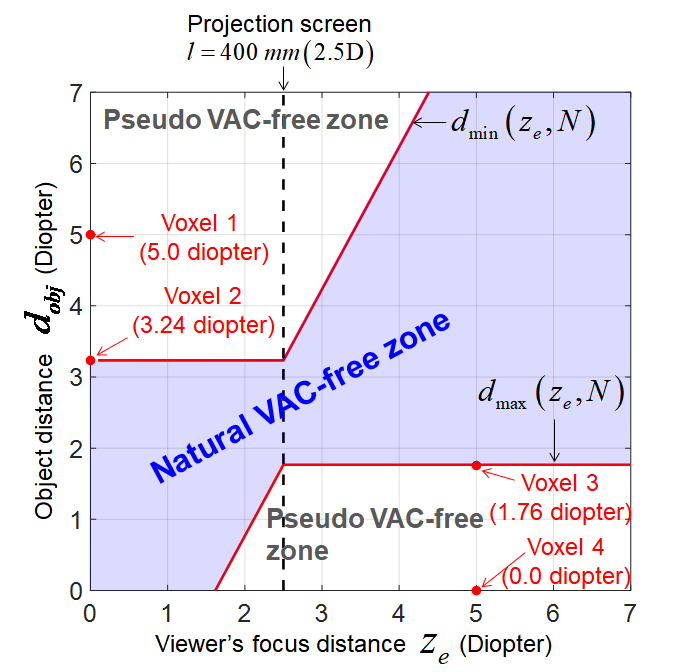


**Figure S9.** Estimation results of the natural-VAC-free zone in the proposed BTS HDSMV projection system by comparing $c_{p}\left(z_{e},N\right)$ and $c_{o}\left(d_{obj},z_{e}\right)$ for a viewer’s focus distance $z_{e}$.

Figure S10 shows and $c_{o}\left(d_{obj},z_{e}\right)$ with respect to $d_{obj}$ for $z_{e}=200 mm$. The green line indicates $c_{o}\left(d_{obj}\text{, }z_{e}=5.0\text{D}\right)$. The voxel with $d_{obj}=200 mm$is in focus, so $c_{o}$is zero. The red line represents $c_{p}\left(z_{e}=5.0\text{D,} N=11\right)$. The intersection of the green and the red lines corresponds to the case where $c_{o}\left(d_{obj}\text{, }z_{e}=5.0\text{D}\right)$ and $c_{p}\left(z_{e}=5.0\text{D,} N=11\right)$ are equal, and the points represents $d_{\max}\left(z_{e}=5.0\text{D},N=11\right)$ and $d_{\min}\left(z_{e}=5.0\text{D},N=11\right)$. The voxels located behind $d_{\max}$ are expected to appear separated because $c_{o}\left(d_{obj}\text{, }z_{e}=5.0\text{D}\right)$ is larger than $c_{p}\left(z_{e}=5.0\text{D,} N=11\right)$. The diffraction-limit of the eye affects $c_{p}\left(z_{e},N\right)$, and $c_{p}\left(z_{e},N\right)$ increases as much as the width of the blue line (Fig. S10). When $z_{e}$ is 200 *mm*, the voxel located at infinity $\left(d_{obj}=\infty\right)$ is separated, but the viewer cannot actually distinguish this separation. Additionally, from Eq. (7), an increase in the number of monocular VRPs $N$ leads to the increase in $c_{p}\left(z_{e},N\right)$. At this time, if the VRP width $w_{v}$ is constant, $c_{p}\left(z_{e},N\right)$ is affected by the diffraction-limit of the eye in accordance with $N$ and $z_{e}$ (Fig. S11). $c_{p}\left(z_{e},N\right)$ increases with $N$, then an increase in the range between $d_{\max}\left(z_{e},N\right)$ and $d_{\min}\left(z_{e},N\right)$ is predicted from Eqs. (10a) and (10b).


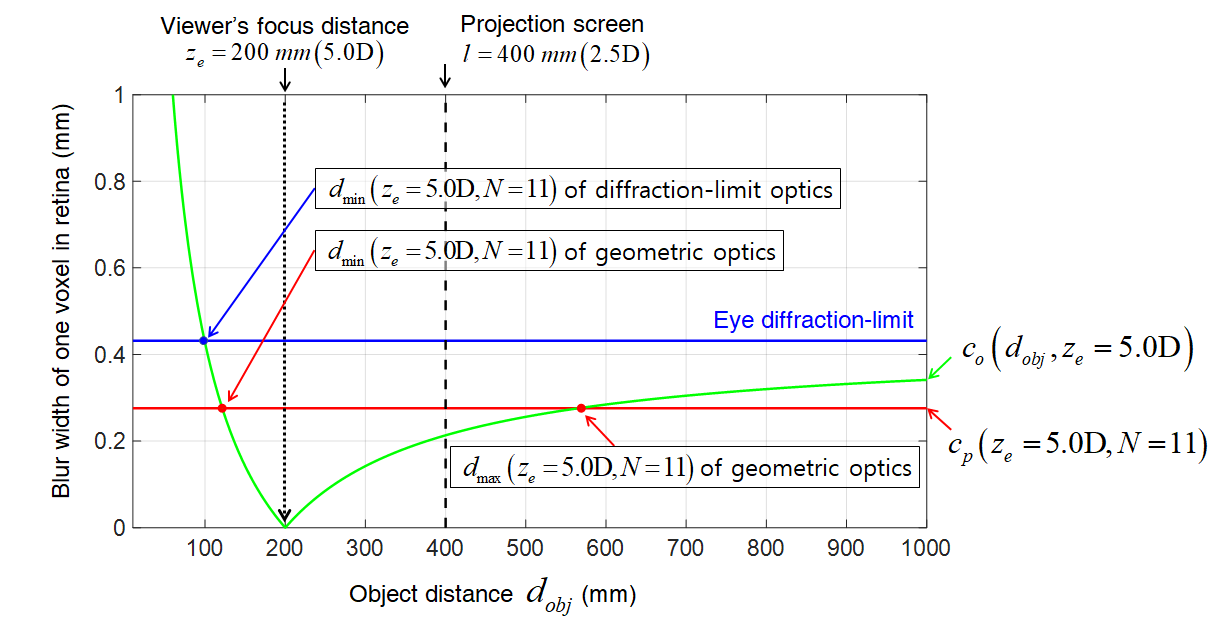


**Figure S10**. Blur width of voxels in the retina according to the object distance $d_{obj}$ for $z_{e}=200 mm$.


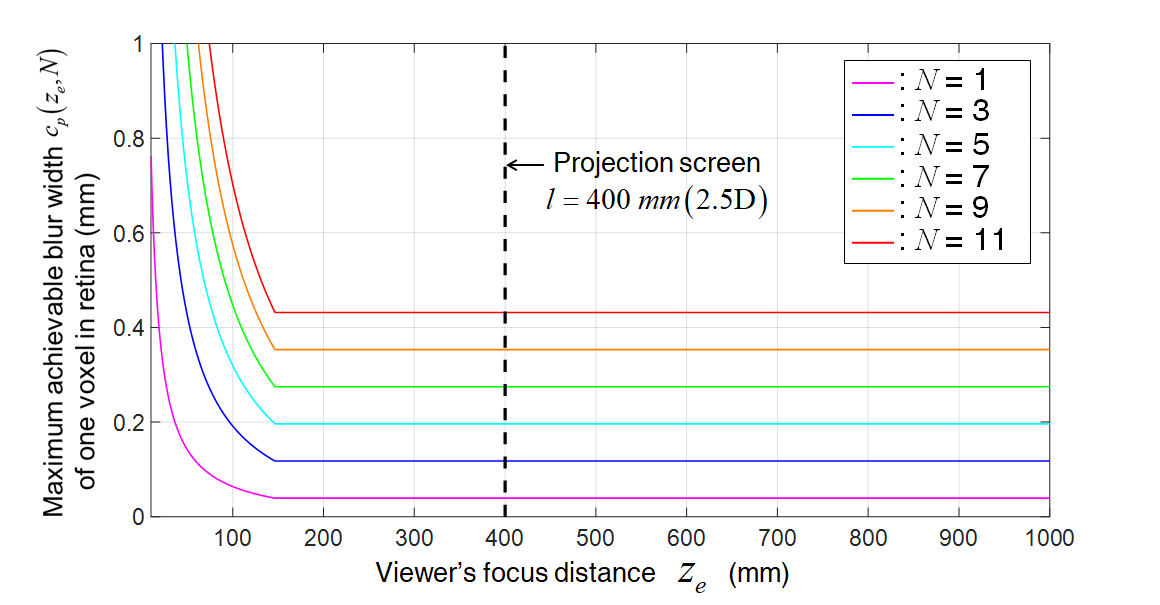


**Figure S11.** Maximum achievable blur width $c_{p}\left(z_{e},N\right)$ of one voxel depending on $N$ and $z_{e}$.

The natural-VAC-free zone is determined by plotting Eqs. (7), (9), (10a), and (10b) with considering the eye-resolution limit (Fig. S12). The red dotted lines in Fig. S12 are $d_{\min}\left(z_{e},N\right)$ and$d_{\max}\left(z_{e},N\right)$, representing the boundaries of the natural VAC-free zone obtained from geometric optic analysis. The red dotted lines are equal to the red lines in Fig. S10. The increase in the range between $d_{\min}\left(z_{e},N\right)$ and $d_{\max}\left(z_{e},N\right)$ due to the eye-resolution limit is explained by comparing the blue lines with the red dotted lines. Voxels 1 and 4 appeared to be separated in the pseudo-VAC-free zone based on geometric optical analysis. However, under the consideration of the diffraction-limit of the eye, voxels 1 and 4 are placed in the natural VAC-free zone. The red zone is the outside of the natural VAC-free zone.


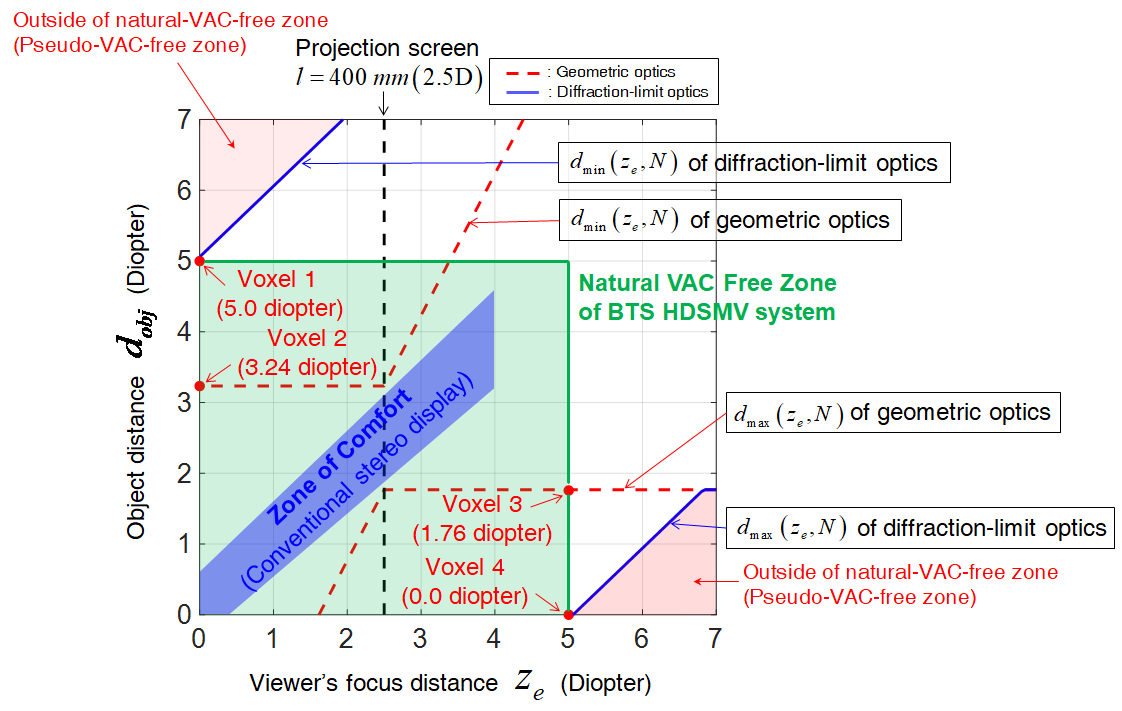


**Figure S12.** Natural VAC-free zone of the proposed BTS HDSMV projection system.

The area satisfying VAC-free condition based on the viewer’s visual perception is represented by the inside between two blue lines where is enclosed by $d_{\min}\left(z_{e}\right)$ and $d_{\max}\left(z_{e}\right)$. Considering the convergence of two eyes and the expressible object distance, the natural VAC-free zone of the proposed BTS HDSMV system is determined as the green rectangular area. This indicates that 3D SMV content positioned at an object distance from 5.0 diopters to 0.0 diopters can be observed without VAC while as $z_{e}$ changes from 5.0 to 0.0 diopters. The proposed BTS HDSMV system thus provides wide-range VAC-free binocular visual perception SMV images. The numerical and experimental results reveal that the proposed BTS HDSMV method widens the natural-VAC-free zone, transcending the limits of previously reported SMV displays by implementing unprecedentedly high SMV density.

**References**

1. T. Shibata, J. Kim, D. M. Hoffman, and M. S. Banks, The zone of comfort: Predicting visual discomfort with stereo displays, *Journal of Vision* **11**, 1-29 (2011)
